# Supplementary material for: Development of a computer-aided design software for the quantitative evaluation of aesthetic damage
Source: PLoS One. 2019 Dec 18;14(12):e0226322. doi: 10.1371/journal.pone.0226322 (PMC6919621; doi:10.1371/journal.pone.0226322)
Supplement: S1 Code site — 1. (ZIP) [file pone.0226322.s001.zip › [EESC_jr][Projeto]Relatorio_07_21/vendors/select2/docs/_includes/options/introduction.html]

This page of the documentation is currently **undergoing a rewrite and may be incomplete**. If you do not find the answer you are looking for on this page, you may have better luck looking at the old options page.

This documentation is set up in the form of a FAQ and covers the most common questions. If you do not find the answer to your question here, you may want to reach out to the community to see if someone else can answer it.
